# Interleukin-21 can efficiently restore impaired antibody-dependent cell-mediated cytotoxicity in patients with oesophageal squamous cell carcinoma

**DOI:** 10.1038/sj.bjc.6605502

**Published:** 2009-12-22

**Authors:** M Watanabe, K Kono, Y Kawaguchi, Y Mizukami, K Mimura, T Maruyama, H Fujii

**Affiliations:** 1 First Department of Surgery, University of Yamanashi, Yamanashi 409-3898, Japan

**Keywords:** IL-21, ADCC, Trastuzumab, Cetuximab

## Abstract

**Background:**

We previously reported that Trastuzumab- and Cetuximab-mediated antibody-dependent cell-mediated cytotoxicity (ADCC) in cancer patients was impaired in comparison with that in healthy donors because of NK-cell dysfunction. In this study, we evaluated whether IL-21 could improve the impairment of ADCC in patients with oesophageal squamous cell carcinoma (ESCC), as IL-21 was reported to have the ability to activate NK cells.

**Methods:**

We examined Trastuzumab- and Cetuximab-mediated ADCC of peripheral blood mononuclear cells (PBMCs) or of enriched NK cells derived from ESCC patients (*n*=20) and healthy donors (*n*=16) in the presence of IL-21. We further analysed ADCC-related molecules (perforin, granzyme-B, and CD247) on NK cells in response to IL-21.

**Results:**

Trastuzumab- and Cetuximab-mediated ADCC of PBMCs or of enriched NK cells was enhanced by the addition of IL-21 in a dose-dependent manner and the levels of ADCC enhanced by IL-21 in patients were high enough in comparison with those in healthy donors, paralleling the upregulation of CD247 on NK cells.

**Conclusion:**

IL-21 could efficiently restore impaired ADCC in ESCC patients with the upregulation of CD247 molecules.

Anti-HER2 mAb, Trastuzumab, was clinically demonstrated to lead to a survival benefit in patients with HER2-overexpressing breast cancer (Slamon *et al*, 2001; Hall and Cameron, 2009). Moreover, anti-HER1 (EGFR) mAb, Cetuximab, has now been approved for use in patients with colorectal cancer (Cunningham *et al*, 2004; Saltz *et al*, 2004). In addition to breast and colon cancer, HER-family overexpression has been identified in a variety of human cancers such as gastrointestinal tract, colorectal, lung, and bladder cancers, and is correlated in a wide variety of tumours with progression (Neal *et al*, 1985; Yarden and Sliwkowski, 2001; Takehana *et al*, 2002; Ooi *et al*, 2004). In particular, we and others have reported that the overexpression of EGFR is present in 50–70% of oesophageal squamous cell carcinoma (ESCC) cases, and is indicative of a poor prognosis (Itakura *et al*, 1994; Hanawa *et al*, 2006). Moreover, we showed the overexpression of HER2 in 30% of ESCC cases (Mimura *et al*, 2005a). These results indicate that ESCC cases show a relatively high incidence of EGFR and/or HER2 overexpression, and that the HER family would be an attractive target for the treatment of ESCC. Furthermore, we showed *in vitro* that HER2- or EGFR-overexpressing ESCC was killed by Trastuzumab- or Cetuximab-mediated antibody-dependent cellular cytotoxicity (ADCC), respectively (Mimura *et al*, 2005b; Kawaguchi *et al*, 2007a, 2007b). Taken together, therapeutic mAbs such as Trastuzumab and Cetuximab targeting the HER family constitute an attractive approach in the treatment of ESCC.

There are many mechanisms that contribute to the anti-tumour activity of Cetuximab and Trastuzumab, including a direct inhibition of tyrosine kinase activity relating to the HER-family signal, the inhibition of cell cycle progression, and increased levels and activities of pro-apoptotic molecules (Sliwkowski *et al*, 1999; Cuello *et al*, 2001). Furthermore, ADCC was proven to be one of the important mechanisms of Trastuzumab, as it was reported that the anti-tumour effects of Trastuzumab were dependent on the presence of Fc receptor-bearing immune cells such as NK cells (Clynes *et al*, 2000). In addition, it was clearly shown in a clinical study that CD16 polymorphism, which is important in ADCC, was significantly correlated with the outcome of Trastuzumab therapy in breast cancer (Varchetta *et al*, 2007).

With regard to ADCC in patients with cancer, we reported that Trastuzumab- and Cetuximab-mediated ADCC was impaired in comparison with healthy donors because of NK-cell dysfunction (Kono *et al*, 2002; Mimura *et al*, 2005b; Kawaguchi *et al*, 2007a). In general, NK cells in cancer-bearing hosts are impaired by many mechanisms, including their reduced number, imbalances in their activating and inhibitory receptor repertoire, as well as immunosuppressive cytokines (Fan *et al*, 1997; Tsuruma *et al*, 1999). Thus, the enhancement of NK-cell function may result in the improvement of impaired ADCC in patients with cancer, leading to successful treatment with Trastuzumab and Cetuximab. For example, immunomodulatory cytokines, including interleukin (IL)-2, IL-12, or IL-21, would be effective adjuvants in the enhancement of impaired ADCC in patients with cancer.

Interleukin-21 is a cytokine that activates CD8 (+) T and NK cells, and those produced by activated CD4 (+) T and NKT cells (Parrish-Novak *et al*, 2000; Coquet *et al*, 2007). Effects of IL-21 include activation of antigen-specific CTLs, activation and differentiation of NK cells, and antibody production (Skak *et al*, 2008b). Furthermore, IL-21 has been shown to be an important autocrine factor of Th17 cells (Nurieva *et al*, 2007). Interleukin-21 belongs to the common *γ*-chain family, with a similarity to IL-2 and IL-21 in its sequence and structure (Asao *et al*, 2001). Although the effect of IL-21 on murine NK cells has been extensively elucidated (Skak *et al*, 2008b), there is still limited information with regard to the effect of IL-21 on human NK cells. There is a report that IL-21 enhanced human NK cytotoxicity against K562, paralleling the upregulation of perforin and granzyme (Skak *et al*, 2008a).

In this study, we evaluate whether IL-21 could improve the impairment of ADCC mediated by Trastuzumab or Cetuximab in patients with ESCC. Moreover, we further analysed the mechanisms behind how IL-21 enhanced ADCC, with particular focus on functional molecules related to the cytotoxicity of NK cells.

## Materials and methods

### Patients

In all, 20 patients with oesophageal squamous cell carcinoma, who were operated on in the University of Yamanashi Hospital from 2008 to 2009, were included in the study. The patients with oesophageal cancer were 73.9±10.3 years old; 19 patients were men and 1 was a women. A total of 6 patients belonged to stage I, 5 were stage II, 8 were stage III, and 1 was stage IV according to the TNM classification for oesophageal cancers. None of the patients received radiotherapy, chemotherapy, or other medical interventions before the study. This study was approved by the ethical committee of the University of Yamanashi, and written informed consent was obtained from all individuals.

### Cell lines

The ESCC cell lines KYSE30, KYSE50, and KYSE110 were purchased from the Health Science Research Resources Bank (Osaka, Japan). The ESCC cell line TE4 was a kind gift from Dr Nishihara (Institute of Development, Aging and Cancer, University of Tohoku, Sendai, Japan). All cells were cultured in RPMI 1640 medium with 5% fetal bovine serum, 100 U ml^−1^ penicillin, 100 *μ*g ml^−1^ streptomycin, and 2 mmol l^−1^ L-glutamine. According to our previous studies (Mimura *et al*, 2005b; Kawaguchi *et al*, 2007a, 2007b), TE4 and KYSE50 were used as high HER2- and low HER2-expressing tumours, respectively, and KYSE30 and KYSE110 were used as high EGFR- and low EGFR-expressing tumours, respectively.

### Chemicals and antibodies

Humanised mouse anti-human EGFR antibody, Cetuximab (Erbitux), was purchased from Merck (Dietikon, Switzerland). The anti-HER-2 monoclonal antibody, Trastuzumab (Herceptin), and anti-CD20 mAb Rituxan, which is an isotype-matched control mAb, were purchased from Roche (Basel, Switzerland). Recombinant human IL-21 was provided by Novo Nordisk (Copenhagen, Denmark). Recombinant human IL-2 was purchased from Peprotech (Rocky Hill, NJ, USA).

### Preparation of cells

Peripheral blood mononuclear cells (PBMCs) were separated from peripheral blood obtained from patients with ESCC and from healthy donors by Ficoll-Paque (Pharmacia, Uppsala, Sweden) density gradient centrifugation.

To prepare NK cells by negative selection, NK cells were isolated from PBMCs by centrifugation with Ficoll-Paque after being incubated with RosetteSep antibody cocktail for NK cells (StemCell Technologies, Vancouver, British Columbia, Canada). The RosetteSep antibody cocktail was bound in bispecific antibody complexes, which are directed against cell-surface antigens on human haematopoietic cells (CD3, CD4, CD19, CD36, and CD66b) and glycophorin A on red blood cells. Unwanted cells, which adhered to red blood cells, and desired cells were separated using a Ficoll-Paque density gradient.

### IL-21 treatment of PBMCs or NK cells

Peripheral blood mononuclear cells (1 × 10^6^ cells ml^−1^) or NK cells (1 × 10^6^ cells ml^−1^) were incubated with X-VIVO medium in 48-well culture plates in the presence or absence of IL-21 for 24 h.

### ADCC assay

After the target cells were labelled with 50 *μ*Ci of ^51^Cr for 60 min, target cells (5 × 10^3^ per well) and PBMCs or NK cells as effector cells were co-incubated at various effector : target ratios in 200 *μ*l of X-VIVO medium in a 96-well U-bottomed plate in triplicate with Trastuzumab (10 *μ*g ml^−1^), Cetuximab (5 *μ*g ml^−1^), or control mAb Rituxan. After 6 h of incubation, radioactivity of the supernatant (100 *μ*l) was measured with a *γ*-counter. The percentage of specific lysis=100 × (experimental c.p.m.−spontaneous c.p.m.)/(maximum c.p.m.−spontaneous c.p.m.).

### Flow cytometry

For the expression of CD247 (transducing *ζ* molecule) on NK cells, PBMCs were stained with CD56-FITC (DAKO, Glostrup, Denmark) and CD3-APC (DAKO). Thereafter, the stained cells were fixed with 0.25% formaldehyde in PBS for 10 min, followed by permeabilisation with digitonin (Invitrogen, Carlsbad, CA, USA; 10 *μ*g ml^−1^) for 15 min on ice, and stained with anti-CD247 mAbs conjugated with PE (IMMUNOTEC, Marseille, France) or with IgG1 isotype control mAbs for 10 min on ice. The mean fluorescence intensity for CD247 gated on CD56(+)CD3(−) cells was assessed by flow cytometric analysis.

For the expression of perforin and granzyme-B on NK cells, PBMCs were stained with CD56-FITC and CD3-APC. Thereafter, the stained cells were fixed and permeabilised using the Cytoperm/Cytofix kit (BD Bioscience, San Diego, CA, USA), followed by staining with mouse anti-human perforin mAbs conjugated with FITC (BD Pharmingen, San Diego, CA, USA) or mouse anti-human Granzyme-B mAbs conjugated with FITC (BD Pharmingen). The mean fluorescence intensity for perforin or granzyme-B gated on CD56(+)CD3(−) cells was assessed by flow cytometric analysis.

For the expression of IL-21 receptor (IL-21R) on NK cells, PBMCs were stained with CD56-FITC, CD3-APC, and PE-labelled anti-human IL-21R mAb (R&D Systems, Minneapolis, MN, USA).

### Statistical analysis

Paired and non-paired Student's *t*-tests were used to examine the differences between groups. Findings were considered significant when *P*-values were <0.05.

## Results

### Trastuzumab- and Cetuximab-mediated ADCC was impaired in patients with ESCC

**Figure 1:**
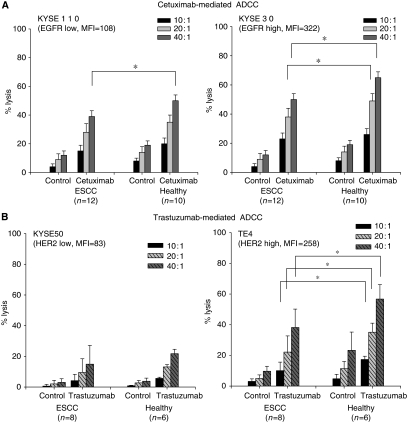
Trastuzumab- and Cetuximab-mediated antibody-dependent cell-mediated cytotoxicity (ADCC) of peripheral blood mononuclear cells (PBMCs) from patients and healthy donors. (**A**) Cetuximab-mediated ADCC of PBMCs from patients (oesophageal squamous cell carcinoma (ESCC), *n*=12) and healthy donors (healthy, *n*=10) was analysed against high EGFR-expressing KYSE30 and low EGFR-expressing KYSE110 cells in the presence of Cetuximab or control mAbs at the indicated effector : target ratios (10 : 1, 20 : 1, and 40 : 1). ^*^P<0.05. (**B**) Trastuzumab-mediated ADCC of PBMCs from patients (ESCC, *n*=8) and healthy donors (healthy, *n*=6) was analysed against high HER2-expressing TE4 and low HER2-expressing KYSE50 cells in the presence of Trastuzumab or control mAbs at the indicated effector : target ratios (10 : 1, 20 : 1, and 40 : 1). ^*^P<0.05. The expression of EGFR or HER2 on target tumour cells was shown as mean fluorescence intensity (MFI) analysed by flow cytometry.

We examined Trastuzumab- and Cetuximab-mediated ADCC of PBMCs derived from patients and healthy donors. High EGFR-expressing KYSE30 and low EGFR-expressing KYSE110 were killed by Cetuximab-mediated ADCC, and the ADCC activity reflected the degree of EGFR expression, as shown in Figure 1A (high EGFR-expressing KYSE30 *vs* low EGFR-expressing KYSE110). It is noteworthy that the levels of Cetuximab-mediated ADCC in patients with ESCC were significantly impaired in comparison with those in healthy donors (Figure 1A), in line with our previous reports (Kawaguchi *et al*, 2007a).

Similarly, HER2-expressing ESCC tumour cells were efficiently killed by Trastuzumab-mediated ADCC, with PBMCs derived from patients and healthy donors, and the ADCC activity reflected the degree of HER2 expression, as shown in Figure 1B (high HER-2-expressing TE4 *vs* low HER-2-expressing KYSE50). It is important to note that the levels of Trastuzumab-mediated ADCC in patients with ESCC were significantly impaired in comparison with those in healthy donors (Figure 1B), in line with our previous report (Mimura *et al*, 2005b).

Taken together, we confirmed that Trastuzumab- and Cetuximab-mediated ADCC was impaired in patients with ESCC in comparison with those in healthy donors, although HER2-expressing or EGFR-expressing ESCC was killed by Trastuzumab- and Cetuximab-mediated ADCC, respectively.

### IL-21 enhanced Cetuximab-mediated ADCC activity of PBMCs derived from ESCC patients

**Figure 2:**
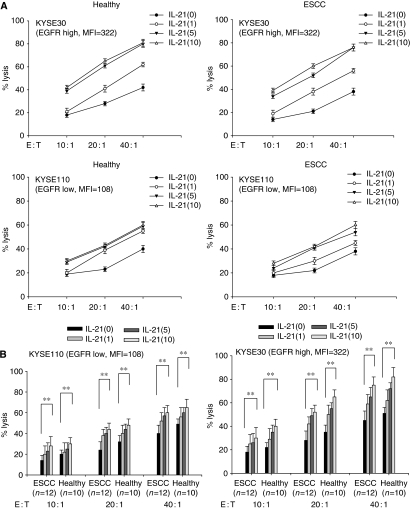
Cetuximab-mediated antibody-dependent cell-mediated cytotoxicity (ADCC) of peripheral blood mononuclear cells (PBMCs) cultured with IL-21. PBMCs derived from patients (oesophageal squamous cell carcinoma (ESCC)) and healthy donors (healthy) were cultured with IL-21 at the indicated doses (1, 5, and 10 *μ*g ml^−1^) for 24 h. Thereafter, cultured PBMCs were subjected to Cetuximab-mediated ADCC against high EGFR-expressing KYSE30 and low EGFR-expressing KYSE110 at the indicated effector : target ratios (10 : 1, 20 : 1, and 40 : 1). (**A**) Representative data showed that Cetuximab-mediated ADCC was enhanced by the addition of IL-21. (**B**) Summarised data from patients (ESCC, *n*=12) and healthy donors (healthy, *n*=10) showed that IL-21 significantly enhanced Cetuximab-mediated ADCC in a dose-dependent manner. ^**^P<0.01. The expression of EGFR on target tumour cells was shown as mean fluorescence intensity (MFI) analysed by flow cytometry.

We next analysed the effect of IL-21 on Cetuximab-mediated ADCC, when PBMCs derived from patients and healthy donors were cultured with IL-21 at indicated doses for 24 h. Representative ADCC data showed that Cetuximab-mediated ADCC activity was significantly enhanced by the addition of IL-21 to PBMCs derived from patients and healthy donors (Figure 2A). Summarised data from patients (*n*=12) and healthy donors (*n*=10) clearly showed that IL-21 significantly enhanced Cetuximab-mediated ADCC against high EGFR-expressing KYSE30 and low EGFR-expressing KYSE110 in a dose-dependent manner (Figure 2B). It is noteworthy that Cetuximab-mediated ADCC enhanced by IL-21 in patients was high enough in comparison with that in healthy donors; for example, 75.1±7.3 *vs* 82.2±8.2%, respectively at an E : T ratio of 40 : 1 and 10 *μ*g ml^−1^ of IL-21 against KYSE30 (Figure 2B), in which the original ADCC levels of patients were significantly impaired in comparison with those of healthy donors (Figure 1A). Thus, IL-21 could efficiently enhance impaired Cetuximab-mediated ADCC in patients with ESCC.

### IL-21 enhanced Trastuzumab-mediated ADCC activity of PBMCs derived from ESCC patients

**Figure 3:**
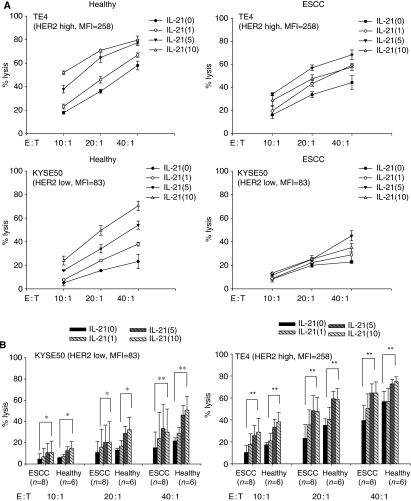
Trastuzumab-mediated antibody-dependent cell-mediated cytotoxicity (ADCC) of peripheral blood mononuclear cells (PBMCs) cultured with IL-21. PBMCs derived from patients (oesophageal squamous cell carcinoma (ESCC)) and healthy donors (healthy) were cultured with IL-21 at the indicated doses (1, 5, and 10 *μ*g ml^−1^) for 24 h. Thereafter, cultured PBMCs were subjected to Trastuzumab-mediated ADCC against high HER2-expressing TE4 and low HER2-expressing KYSE50 at the indicated effector : target ratios (10 : 1, 20 : 1, and 40 : 1). (**A**) Representative data showed that Trastuzumab-mediated ADCC was enhanced by the addition of IL-21. (**B**) Summarised data from patients (ESCC, *n*=8) and healthy donors (healthy, *n*=6) showed that IL-21 significantly enhanced Trastuzumab-mediated ADCC in a dose-dependent manner. ^**^P<0.01. ^*^P<0.05. The expression of HER2 on target tumour cells was shown as mean fluorescence intensity (MFI) analysed by flow cytometry.

We further investigated the effect of IL-21 on Trastuzumab-mediated ADCC, when PBMCs derived from patients and healthy donors were cultured with IL-21 at indicated doses for 24 h. Representative ADCC data indicated that Trastuzumab-mediated ADCC activity was significantly enhanced by the addition of IL-21 to PBMCs derived from patients and healthy donors (Figure 3A). Summarised data from patients (*n*=8) and healthy donors (*n*=6) clearly showed that IL-21 could significantly enhance Trastuzumab-mediated ADCC activity against high HER2-expressing TE4 and low HER2-expressing KYSE50 in a dose-dependent manner (Figure 3B). It is important to note that the ADCC enhanced by IL-21 in patients was high enough in comparison with that in healthy donors; for example, 62.7±10.5 *vs* 74.9±4.3%, respectively at an E : T ratio of 40 : 1 and 10 *μ*g ml^−1^ of IL-21 against TE4 (Figure 3B), in which the original ADCC levels in patients were significantly impaired in comparison with those in healthy donors (Figure 1B). Thus, the enhancement by IL-21 of impaired ADCC in patients was also confirmed with regard to Trastuzumab-mediated ADCC.

### IL-21 enhanced ADCC mediated by enriched NK cells

**Figure 4:**
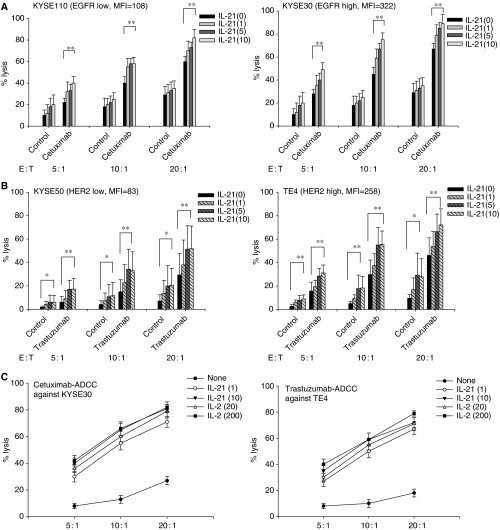
Cetuximab- and Trastuzumab-mediated antibody-dependent cell-mediated cytotoxicity (ADCC) of NK cells cultured with IL-21. Purified NK cells derived from healthy donors (*n*=7) were cultured with IL-21 at indicated doses (1, 5, and 10 *μ*g ml^−1^) for 24 h. Thereafter, cultured NK cells were subjected to an ADCC assay against high EGFR-expressing KYSE30 and low EGFR-expressing KYSE110 at the indicated effector : target ratios (5 : 1, 10 : 1, and 20 : 1) in the presence of Cetuximab or control mAbs (Control, **A**). Similarly, the cultured NK cells were subjected to an ADCC assay against high HER2-expressing TE4 and low HER2-expressing KYSE50 at the indicated effector : target ratios (5 : 1, 10 : 1, and 20 : 1) in the presence of Trastuzumab or control mAbs (Control, **B**). ^**^P<0.01. ^*^P<0.05. The expression of HER2 or EGFR on target tumour cells was shown as mean fluorescence intensity (MFI) analysed by flow cytometry. In (**C**), purified NK cells derived from healthy donors were cultured with IL-21 (1 and 10 *μ*g ml^−1^) or IL-2 (20 and 200 ng ml^−1^) for 24 h. The cultured NK cells were subjected to an ADCC assay against high HER2-expressing TE4 or high EGFR-expressing KYSE30 at the indicated effector : target ratios (5 : 1, 10 : 1, and 20 : 1) in the presence of Trastuzumab or Cetuximab. Representative ADCC assay from three different experiments from healthy donors (*n*=3) was shown in (**C**).

As the use of purified NK cells *vs* PBMC cultures might influence the effect of IL-21 because of the presence of accessory cells, we further analysed the effect of IL-21 on ADCC mediated by NK cells, when enriched NK cells were cultured with IL-21 at indicated doses for 24 h. NK cells from healthy donors (*n*=7) were enriched using a negative selection kit and were confirmed to be more than 93% positive for CD56(+)CD3(−) by flow cytometry. Summarised data showed that Cetuximab-mediated ADCC of NK cells was enhanced by the addition of IL-21 against both high EGFR- and low EGFR-expressing ESCC (Figure 4A). Similarly, Trastuzumab-mediated ADCC of NK cells was enhanced by the addition of IL-21 against both high HER2- and low HER2-expressing ESCC (Figure 4B). These results indicated that IL-21 directly affected NK cells, leading to ADCC enhancement.

Furthermore, the enhancement of ADCC induced by IL-21 was compared with those induced by IL-2. Enriched NK cells from healthy donors (*n*=3) were cultured with IL-21 or IL-2 at indicated doses for 24 h and subjected to ADCC assay. As a result, Cetuximab-mediated ADCC against high EGFR-expressing KYSE30 induced by IL-21 (1 and 10 *μ*g ml^−1^) was comparable to those induced by IL-2 (20 and 200 ng ml^−1^) (Figure 4C). Similarly, Trastuzumab-mediated ADCC against high HER2-expressing TE4 was comparable to those induced by IL-2 (Figure 4C). This observation was confirmed in three different experiments with NK cells from different healthy donors (*n*=3).

### Alteration of ADCC-related molecules on NK cells in response to IL-21

**Figure 5:**
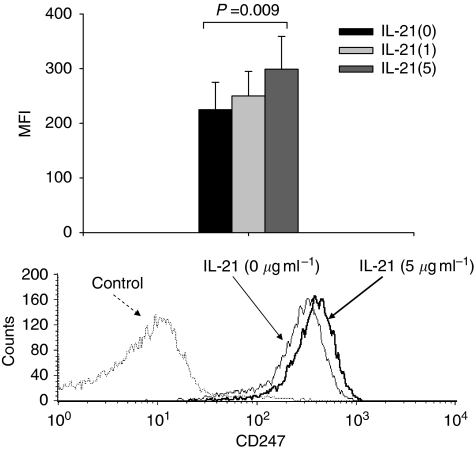
Upregulation of CD247 on NK cells in response to IL-21. Peripheral blood mononuclear cells (PBMCs) derived from patients (*n*=12) were cultured with IL-21 at the indicated doses (1 and 5 *μ*g ml^−1^) for 24 h. Thereafter, the expression of CD247 molecules on NK cells (CD56(+)CD3(−)) was analysed by intracellular staining with flow cytometry. Representative data of flow cytometry and summarised data (*n*=12) indicated that the expression of CD247 on NK cells was significantly enhanced by treatment with IL-21 at 5 *μ*g ml^−1^.

We further analysed how IL-21 enhances ADCC with particular focus on ADCC-related molecules on NK cells. As it has been reported that CD247 molecules (signal-transducing *ζ* molecules) on NK cells were related to CD16 (Fc receptor)-related cytotoxicity (Whiteside, 2004), we evaluated the expression of CD247 molecules on NK cells (CD56(+)CD3(−)), analysed by intracellular staining with flow cytometry, when PBMCs in patients with ESCC were treated with IL-21. Representative flow cytometric and summarised data (*n*=12) indicated that the expression of CD247 on NK cells was significantly enhanced by treatment with IL-21 at 5 *μ*g ml^−1^ (Figure 5).

**Figure 6:**
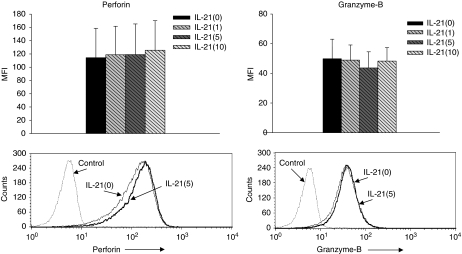
Perforin and granzyme-B on NK cells cultured with IL-21. Peripheral blood mononuclear cells (PBMCs) derived from patients (*n*=8) were cultured with IL-21 at the indicated doses (1, 5, and 10 *μ*g ml^−1^) for 24 h. Thereafter, the expression of perforin and granzyme-B molecules on NK cells (CD56(+)CD3(−)) was analysed by intracellular staining with flow cytometry. Representative data of flow cytometry and summarised data are shown.

Next, as it has been shown that IL-21 increased perforin and granzyme-B (Skak *et al*, 2008a), we evaluated their expression on NK cells by intracellular staining with flow cytometry, when PBMCs (*n*=8) from patients were treated with IL-21. As shown in Figure 6, the expression of perforin or granzyme-B on NK cells was not significantly enhanced by the addition of IL-21 in the present setting.

### Expression of IL-21 receptor on NK cells

**Figure 7:**
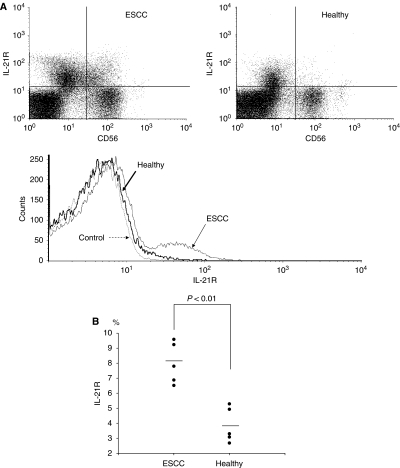
Expression of IL-21 receptor on NK cells. Peripheral blood mononuclear cells (PBMCs) were stained with anti-IL-21 receptor (IL-21R) mAb in combination with anti-CD56 and anti-CD3 mAbs, and the percentage of IL-21R(+) cells gated on CD56(+)CD3(−) cells was analysed by flow cytometry. Representative flow cytometric data are shown in (**A**). Summarised data from healthy donors (healthy, *n*=5) and oesophageal squamous cell carcinoma (ESCC) patients (*n*=5) are shown in (**B**).

Peripheral blood mononuclear cells were stained with anti-IL-21 receptor (IL-21R) mAbs in combination with anti-CD56 and anti-CD3 mAbs, and the percentage of IL-21R(+) cells gated on CD56(+)CD3(−) cells was analysed by flow cytometry. Representative flow cytometric data indicated that the frequency of IL-21R-positive NK cells in a patient with ESCC was increased in comparison with that in a healthy donor (Figure 7A). Summarised data from healthy donors (*n*=5) and ESCC patients (*n*=5) showed that IL-21R-positive NK cells were significantly increased in ESCC patients compared with healthy donors (Figure 7B).

## Discussion

This study provided important and novel findings that IL-21 could efficiently restore impaired ADCC in ESCC patients with the upregulation of CD247 molecules.

There is increasing evidence that ADCC is one of the important mechanisms behind therapeutic mAbs such as Trastuzumab and Cetuximab, which have an anti-tumour effect (Clynes *et al*, 2000; Varchetta *et al*, 2007). However, we and others reported that ADCC activity was downregulated in patients with cancer, mainly because of NK-cell dysfunction, compared with that in healthy donors (Kono *et al*, 2002; Mimura *et al*, 2005b; Kawaguchi *et al*, 2007a). Thus, ADCC enhancement in patients may lead to successful treatment with therapeutic mAbs such as Trastuzumab and Cetuximab. For example, immunomodulatory cytokines including IL-2, IL-12, or IL-21 would be effective adjuvants in the enhancement of impaired ADCC in patients with cancer. In a previous study, IL-21 was reported to have the ability to enhance ADCC or NK activity in human *in vitro* models (Skak *et al*, 2008a). In this study, we provided clear evidence, for the first time, that IL-21 could efficiently restore impaired ADCC mediated by Trastuzumab and Cetuximab in patients with ESCC. These results encourage us to apply combination therapy of therapeutic mAbs with IL-21.

Although the effect of IL-21 on murine NK cells has been extensively elucidated, there is still limited information about the effect of IL-21 on human NK cells (Skak *et al*, 2008b). IL-21 had only moderate effects on surface levels of inhibitory (CD158a and CD158b) and activating NK-cell receptors (NKG2D) in human NK cells (Skak *et al*, 2008a). Moreover, IL-21 was able to increase both mRNA and protein levels of the effector molecules perforin and granzyme-B, as well as enhance NK cytotoxicity against K562 (Skak *et al*, 2008a). In this study, we provided novel findings that IL-21 induced the significant upregulation of CD247 molecules on NK cells, important molecules for Fc receptor (CD16)-dependent NK killing (Whiteside, 2004). These results suggested that the upregulation of CD247 molecules was one of the mechanisms behind IL-21-enhanced ADCC in patients with ESCC. However, we did not detect the upregulation of perforin and granzyme-B in response to IL-21 treatment in this study. Although the reason for this discrepancy is currently unknown, it may be due to the difference in *in vitro* culture condition, for example, unfractionated PBMCs *vs* purified NK cells, or incubation time.

In this study, we showed that IL-21 could directly act on NK cells, as one of the mechanisms behind IL-21 enhances ADCC activity, as it was shown that purified NK cells treated with IL-21 could enhance ADCC activity. In addition, it was previously shown that IL-21 indirectly enhanced NK cell function through cytokine production such as IFN-*γ*, when PBMCs were treated with IL-21 (Roda *et al*, 2007). Thus, it is likely that IL-21 has pleiotrophic roles in a wide variety of cells, leading to the enhancement of ADCC activity (Roda *et al*, 2006).

In this study, we showed that the highest dose of IL-21 was sometimes less effective for the enhancement of ADCC than IL-21 at lower levels. It is likely that there might be some plateau effect of IL-21 at the highest dose in some patients, although further experiments are needed to clarify the mechanisms.

A phase I study involving IL-21 monotherapy for metastatic melanoma or renal cell carcinoma reported that monotherapy was well tolerated and exhibited anti-tumour activity in some patients (Davis *et al*, 2007; Thompson *et al*, 2008), thereby suggesting that IL-21 may have an anti-tumour effect as a monotherapy. However, this study clearly showed that IL-21 could efficiently enhance impaired ADCC activity in ESCC patients, suggesting that combination therapy of Trastuzumab or Cetuximab with IL-21 might result in the enhancement of the anti-tumour effect. Furthermore, it was previously shown that the combination of IL-2 and IL-21 could induce additional effects on the enhancement of ADCC activity (Skak *et al*, 2008a). Thus, immunomodulatory cytokines including IL-2, IL-12, or IL-21 would be effective adjuvants in the enhancement of impaired ADCC in patients with cancer.

Regarding the IL-21R on NK cells, we showed in this study that IL-21R-positive NK cells were significantly increased in ESCC patients than in healthy donors. This observation indicated that IL-21 is capable of inducing NK-cell activation in patients with ESCC. Furthermore, the observation for upregulated IL-21R was also found in NK cells of patients with inflammatory bowel disease (IBD) (Liu *et al*, 2009), suggesting that the expression of IL-21R on NK cells may be upregulated in response to chronic inflammatory reactions such as IBD or ESCC. The response to IL-21 is also affected by a polymorphism in the IL-21R gene (Pène *et al*, 2006). Moreover, dimorphism in the gene encoding Fc*γ*RIIIa influences the binding affinity between the Fc receptor (CD16) and mAbs (Wu *et al*, 1997). These observations suggest that genetic factors might have an important role in determining the clinical efficacy of therapeutic mAbs when a cytokine adjuvant is used. In this study, the levels of IL-21-enhanced ADCC mediated by PBMCs did not markedly differ among patients or healthy donors. However, it may be important to evaluate genetic factors such as polymorphism of the IL-21R gene or Fc*γ*RIIIa in patients when clinical trials with mAbs in combination with IL-21 are initiated.

In conclusion, IL-21 could efficiently restore impaired ADCC in patients with ESCC, suggesting that the combination therapy of Trastuzumab or Cetuximab with IL-21 might lead to an enhancement of the anti-tumour effect.
